# Supplementary material for: Heparin-based hydrogel scaffolding alters the transcriptomic profile and increases the chemoresistance of MDA-MB-231 triple-negative breast cancer cells
Source: Biomater Sci. 2020 Feb 13;8(10):2786–96. doi: 10.1039/c9bm01481k (PMC7497406; doi:10.1039/c9bm01481k)
Supplement: Supplementary file 2 [file BM-008-C9BM01481K-s002.zip › Supplementary File 4/HP-B hydrogel + EGF (s)vControl/Pathways/my_analysis.Gsea.1545201170987/HALLMARK_MYC_TARGETS_V2.html]

Details for gene set HALLMARK\_MYC\_TARGETS\_V2[GSEA]

|  || Dataset | expr.class.cls#HYDEGF\_versus\_CONTROL.class.cls#HYDEGF\_versus\_CONTROL\_repos |
| Phenotype | class.cls#HYDEGF\_versus\_CONTROL\_repos |
| Upregulated in class | HYDEGF |
| GeneSet | HALLMARK\_MYC\_TARGETS\_V2 |
| Enrichment Score (ES) | 0.50167894 |
| Normalized Enrichment Score (NES) | 1.8701993 |
| Nominal p-value | 0.0 |
| FDR q-value | 2.368421E-4 |
| FWER p-Value | 0.001 |
Table: GSEA Results Summary

  

Fig 1: Enrichment plot: HALLMARK\_MYC\_TARGETS\_V2      
 Profile of the Running ES Score & Positions of GeneSet Members on the Rank Ordered List

  

| PROBE | DESCRIPTION (from dataset) | GENE SYMBOL | GENE\_TITLE | RANK IN GENE LIST | RANK METRIC SCORE | RUNNING ES | CORE ENRICHMENT || 1 | PPRC1 | na |  |  | 127 | 2.801 | 0.0579 | Yes |
| 2 | NOP2 | na |  |  | 279 | 2.413 | 0.1055 | Yes |
| 3 | RRP12 | na |  |  | 389 | 2.244 | 0.1515 | Yes |
| 4 | TCOF1 | na |  |  | 441 | 2.184 | 0.1991 | Yes |
| 5 | GRWD1 | na |  |  | 633 | 1.975 | 0.2346 | Yes |
| 6 | MYBBP1A | na |  |  | 667 | 1.933 | 0.2774 | Yes |
| 7 | NOP56 | na |  |  | 837 | 1.790 | 0.3098 | Yes |
| 8 | PUS1 | na |  |  | 936 | 1.726 | 0.3444 | Yes |
| 9 | SLC19A1 | na |  |  | 985 | 1.698 | 0.3809 | Yes |
| 10 | MRTO4 | na |  |  | 2344 | 1.166 | 0.3368 | Yes |
| 11 | PLK4 | na |  |  | 2424 | 1.146 | 0.3591 | Yes |
| 12 | MCM5 | na |  |  | 2694 | 1.082 | 0.3700 | Yes |
| 13 | NOC4L | na |  |  | 2758 | 1.062 | 0.3911 | Yes |
| 14 | TBRG4 | na |  |  | 3040 | 0.995 | 0.3993 | Yes |
| 15 | MCM4 | na |  |  | 3125 | 0.976 | 0.4174 | Yes |
| 16 | RRP9 | na |  |  | 3207 | 0.957 | 0.4352 | Yes |
| 17 | SRM | na |  |  | 3681 | 0.869 | 0.4305 | Yes |
| 18 | MAP3K6 | na |  |  | 3822 | 0.842 | 0.4426 | Yes |
| 19 | AIMP2 | na |  |  | 4042 | 0.805 | 0.4497 | Yes |
| 20 | SLC29A2 | na |  |  | 4230 | 0.777 | 0.4578 | Yes |
| 21 | MYC | na |  |  | 4314 | 0.764 | 0.4711 | Yes |
| 22 | PPAN | na |  |  | 4393 | 0.751 | 0.4843 | Yes |
| 23 | BYSL | na |  |  | 4524 | 0.731 | 0.4943 | Yes |
| 24 | UNG | na |  |  | 4743 | 0.698 | 0.4990 | Yes |
| 25 | WDR74 | na |  |  | 4981 | 0.654 | 0.5017 | Yes |
| 26 | NOLC1 | na |  |  | 5664 | 0.551 | 0.4787 | No |
| 27 | UTP20 | na |  |  | 6202 | 0.475 | 0.4616 | No |
| 28 | PRMT3 | na |  |  | 6486 | 0.437 | 0.4569 | No |
| 29 | DCTPP1 | na |  |  | 6695 | 0.412 | 0.4555 | No |
| 30 | NOP16 | na |  |  | 6819 | 0.395 | 0.4582 | No |
| 31 | RABEPK | na |  |  | 7246 | 0.338 | 0.4437 | No |
| 32 | PLK1 | na |  |  | 7470 | 0.309 | 0.4392 | No |
| 33 | IPO4 | na |  |  | 7543 | 0.299 | 0.4423 | No |
| 34 | GNL3 | na |  |  | 8823 | 0.144 | 0.3788 | No |
| 35 | PES1 | na |  |  | 9112 | 0.112 | 0.3663 | No |
| 36 | TFB2M | na |  |  | 9319 | 0.086 | 0.3576 | No |
| 37 | IMP4 | na |  |  | 10094 | 0.007 | 0.3173 | No |
| 38 | EXOSC5 | na |  |  | 10294 | -0.021 | 0.3074 | No |
| 39 | WDR43 | na |  |  | 10309 | -0.024 | 0.3072 | No |
| 40 | SORD | na |  |  | 10833 | -0.083 | 0.2818 | No |
| 41 | LAS1L | na |  |  | 10967 | -0.101 | 0.2772 | No |
| 42 | FARSA | na |  |  | 11456 | -0.155 | 0.2553 | No |
| 43 | RCL1 | na |  |  | 11478 | -0.160 | 0.2579 | No |
| 44 | DDX18 | na |  |  | 11492 | -0.163 | 0.2609 | No |
| 45 | CDK4 | na |  |  | 12236 | -0.251 | 0.2279 | No |
| 46 | SUPV3L1 | na |  |  | 12310 | -0.262 | 0.2301 | No |
| 47 | MPHOSPH10 | na |  |  | 12493 | -0.285 | 0.2272 | No |
| 48 | NIP7 | na |  |  | 12592 | -0.297 | 0.2289 | No |
| 49 | TMEM97 | na |  |  | 12724 | -0.318 | 0.2294 | No |
| 50 | PA2G4 | na |  |  | 13412 | -0.405 | 0.2028 | No |
| 51 | CBX3 | na |  |  | 13745 | -0.448 | 0.1958 | No |
| 52 | HSPD1 | na |  |  | 14479 | -0.552 | 0.1702 | No |
| 53 | HSPE1 | na |  |  | 15347 | -0.696 | 0.1409 | No |
| 54 | HK2 | na |  |  | 15926 | -0.806 | 0.1293 | No |
| 55 | PHB | na |  |  | 15933 | -0.807 | 0.1476 | No |
| 56 | NDUFAF4 | na |  |  | 16686 | -0.996 | 0.1312 | No |
Table: GSEA details [plain text format]

  

Fig 2: HALLMARK\_MYC\_TARGETS\_V2      
 Blue-Pink O' Gram in the Space of the Analyzed GeneSet

  

Fig 3: HALLMARK\_MYC\_TARGETS\_V2: Random ES distribution      
 Gene set null distribution of ES for **HALLMARK\_MYC\_TARGETS\_V2**
